# (3*S*,6*E*)-nerolidol-mediated rendezvous of *Cyclocephala paraguayensis* beetles in bottle gourd flowers

**DOI:** 10.1371/journal.pone.0235028

**Published:** 2020-12-23

**Authors:** Arodí P. Favaris, Amanda C. Túler, Weliton D. Silva, Sérgio R. Rodrigues, Walter S. Leal, José M. S. Bento

**Affiliations:** 1 Department of Entomology and Acarology, “Luiz de Queiroz” College of Agriculture, University of São Paulo (USP), Piracicaba, São Paulo, Brazil; 2 State University of Mato Grosso do Sul, Cassilândia, Mato Grosso do Sul, Brazil; 3 Department of Molecular and Cellular Biology, University of California, Davis, California, United States of America; Ghent University, BELGIUM

## Abstract

Cyclocephalini beetles of the genus *Cyclocephala* (Coleoptera: Melolonthidae: Dynastinae) use flowers of some plants as food, shelter, and mating sites. However, little is known about floral scent chemistry involved in this interaction. Here we show that a sesquiterpene alcohol mediates attraction of *Cyclocephala paraguayensis* Arrow, on bottle gourd flowers, *Lagenaria siceraria* (Cucurbitaceae). Both males and females started to aggregate on the flowers at twilight; after that, mating began and remained for the entire night. GC-FID/EAD analysis of the *L*. *siceraria* floral scent collected in the field revealed that only the major constituent of the airborne volatiles elicited electroantennographic responses on male and female antennae of *C*. *paraguayensis*. This compound was identified as *(*3*S*,6*E)*-nerolidol, which was tested in two field trapping trials in Brazil. In the first bioassay, traps baited with nerolidol (mix of isomers) captured significantly more adult *C*. *paraguayensis* than control traps. In the second field trial, catches in traps baited with a mixture of isomers or enantiopure nerolidol were significantly higher than captures in control traps, but the treatments did not differ significantly. Analysis from the gut content of adult *C*. *paraguayensis* showed the presence of pollen, suggesting that they also use bottle gourd flowers for their nourishment. Taken together, these results suggest that *(*3*S*,6*E)*-nerolidol plays an essential role in the reproductive behavior of *C*. *paraguayensis* by eliciting aggregation, mating, and feeding.

## Introduction

Floral scents have been reported as essential assets for the visitation of beetles of the tribe Cyclocephalini (Melolonthidae: Dynastinae) [1–4]. The interaction of beetles and flowers may result in mutualistic pollination typically by the cantharophilous floral syndrome of basal angiosperms, whose flowers are usually bisexual, protogynous, very scented, and thermogenic [4, 5]. The Cyclocephalini beetles are primarily attracted by strong odors released by flowers as chemical cues to indicate a rendezvous site for food, shelter, and mating sites [4, 6, 7].

The floral volatile organic compounds (VOCs) in the plant families of Araceae, Annonaceae, Arecaceae, and Magnoliaceae, have been demonstrated to be comprised of pyrazines, ketones, esters, and methoxystyrenes [8–13]. The qualitative chemical profile of theses VOCs has shown little complexity, dominated by uncommon and non-related compounds that are sufficient to ensure the attraction of beetles, which display an efficient olfactory recognition that evokes a specific plant-pollinator interaction [13, 14].

By contrast, VOCs produced by eudicot flowers that are visited by Cyclocephalini beetles of the genus *Cyclocephala* have never been identified. It has been suggested that some cyclocephalines of the genus *Cyclocephala* are opportunistic eudicot flower visitors, causing at times damages on flower tissues due to feeding and, therefore, compromising the reproductive capability of the plant [3]. However, this hypothesized unspecific attraction is still poorly understood. It has been hypothesized that the detection of floral odors of eudicot plants by *Cyclocephala* beetles could be an acquired preference from larval feeding habitat in the case of roots having a taste similar to that of flowers, thus, preconditioning insects to the adult stage [15]. Alternately, Cyclocephala beetles might visit eudicot plants because flowers, which could be releasing compounds commonly found in insect volatiles, as preconized by the hypothesis of pre-existing sensory bias [16, 17]. That means the beetles have developed olfactory sensibility to recognize floral scents which are based on chemicals already produced by the insects for intraspecific communication.

*Cyclocephala paraguayensis* Arrow, is a Neotropical species that is frequently found on eudicot plant species [15, 18]. Here, we observed this beetle visiting flowers of *Lagenaria siceraria* (Molina) Standle (Cucurbitaceae), which incidentally represents a new host flower record for the beetle family Melolonthidae. This plant is originated from Africa and is now spread worldwide [19]; it produces white flowers with separate sexes in the same plant (monoecious) but can assume andromonoecious form when hermaphrodite flowers are present [20]. Anthesis occurs throughout the night, at which time lepidopteran pollinators, specially hawkmoths, visit the flowers [21].

We surmised that the volatile components of bottle gourd flowers mediate aggregative behavior of *C*. *paraguayensis* for feeding and mating. We studied the floral scent chemistry of *L*. *siceraria* flowers and showed that *(*3*S*,6*E)*-nerolidol plays an essential role in the reproductive behavior of *C*. *paraguayensis* by eliciting aggregation, mating, and feeding.

## Results

### Sexual and feeding behavior of insects

The behavior of *C*. *paraguayensis* visitation on *L*. *siceraria* flowers was observed under field conditions at night for four hours (7:00 p.m. to 11:00 p.m.). Beetles activity started by the twilight (~7:30 p.m.), when males of *C*. *paraguayensis* were the first to land on *L*. *siceraria* flowers (n = 36; two-tailed Exact Binomial test; *P* = 0.011). At 8:00 p.m., the number of visiting males and females was 18.50 ± 5.97 (mean ± SD), increasing until 11:00 p.m. to 26.75 ± 7.46. Paired males and females of *C*. *paraguayensis* (2.25 ± 3.2) were present on bottle gourd flowers from 8:00 p.m. until the end of observation period. Both sexes of *L*. *siceraria* flowers were visited. Seventy-five percent of bottle gourd flowers harbored more than one mating couple, thus, suggesting that aggregation on bottle gourd flowers created mating opportunities for the beetles.

**Fig 1:**
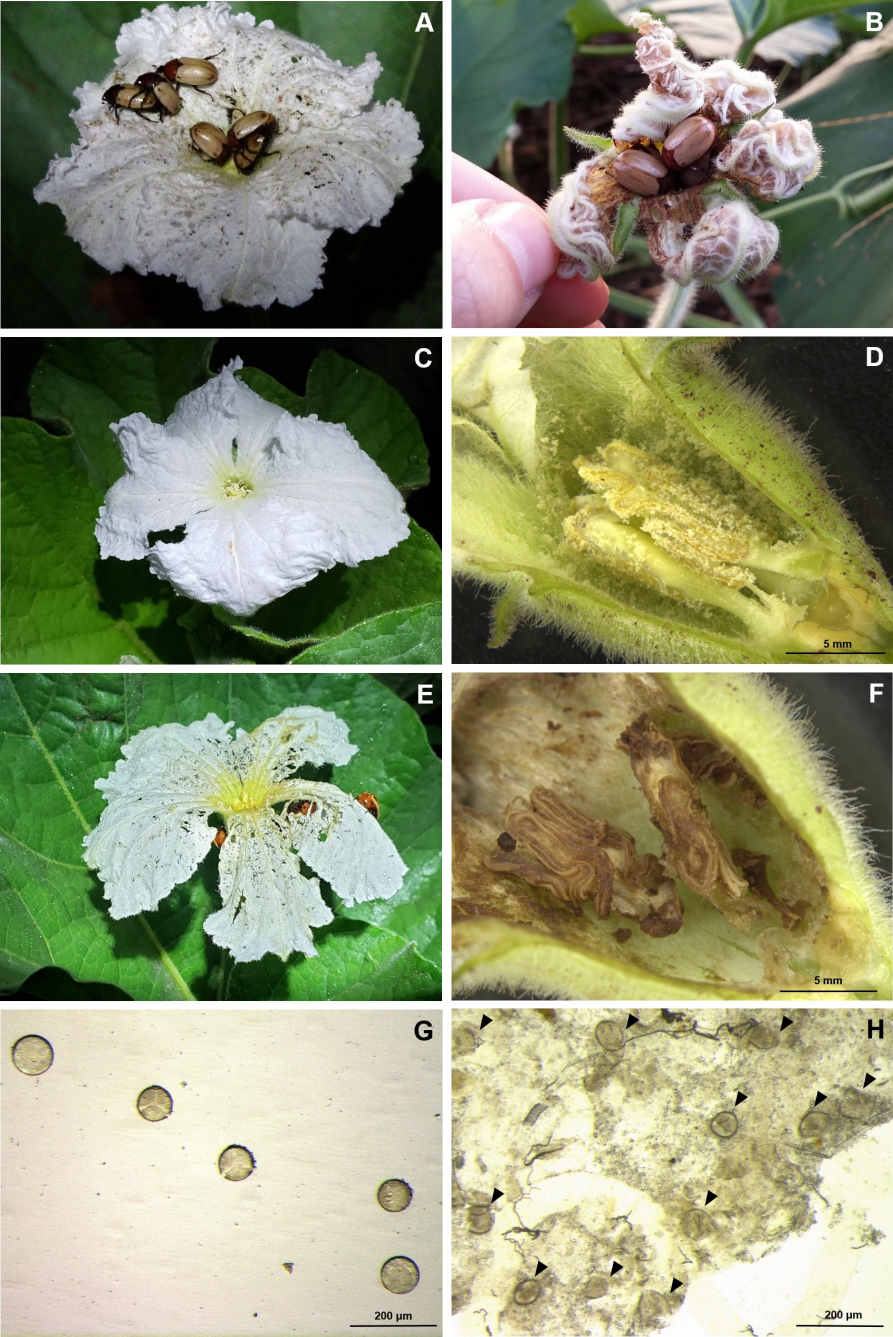
Sexual and feeding behavior of Cyclocephalini beetle, *Cyclocephala paraguayensis*, in bottle gourd flowers, *Lagenaria siceraria*. A) aggregating and mating on flowers at night; B) *C*. *paraguayensis* inside senescent flowers in the next morning of beetle´s attraction; C) Non-damaged *L*. *siceraria* flower in the field; D) Stereoscopic cut view of a non-damaged *L*. *siceraria* flower; E) *L*. *siceraria* flower damaged by *C*. *paraguayensis* in the field; F) Stereoscopic cut view of a damaged flower showing beetle gnawing and excrement signal as a result of feeding activity; G) Fresh pollen grain of *L*. *siceraria*; H) Pollen grains of *L*. *siceraria* extracted from *C*. *paraguayensis* gut (black triangles indicate the pollen grains).

These Cyclocephalini beetles usually remained inside the floral receptacles until the next morning, when they were sheltered (Fig 1A and 1B). Bottle gourd flowers presented gnawing marks or even major damages resulted from beetles feeding (Fig 1E and 1F). Of note, pollens of these flowers were found in the gut of beetles (80% females; 50% males, n = 10) when dissected (Fig 1G and 1H).

### Floral scent identification

**Fig 2:**
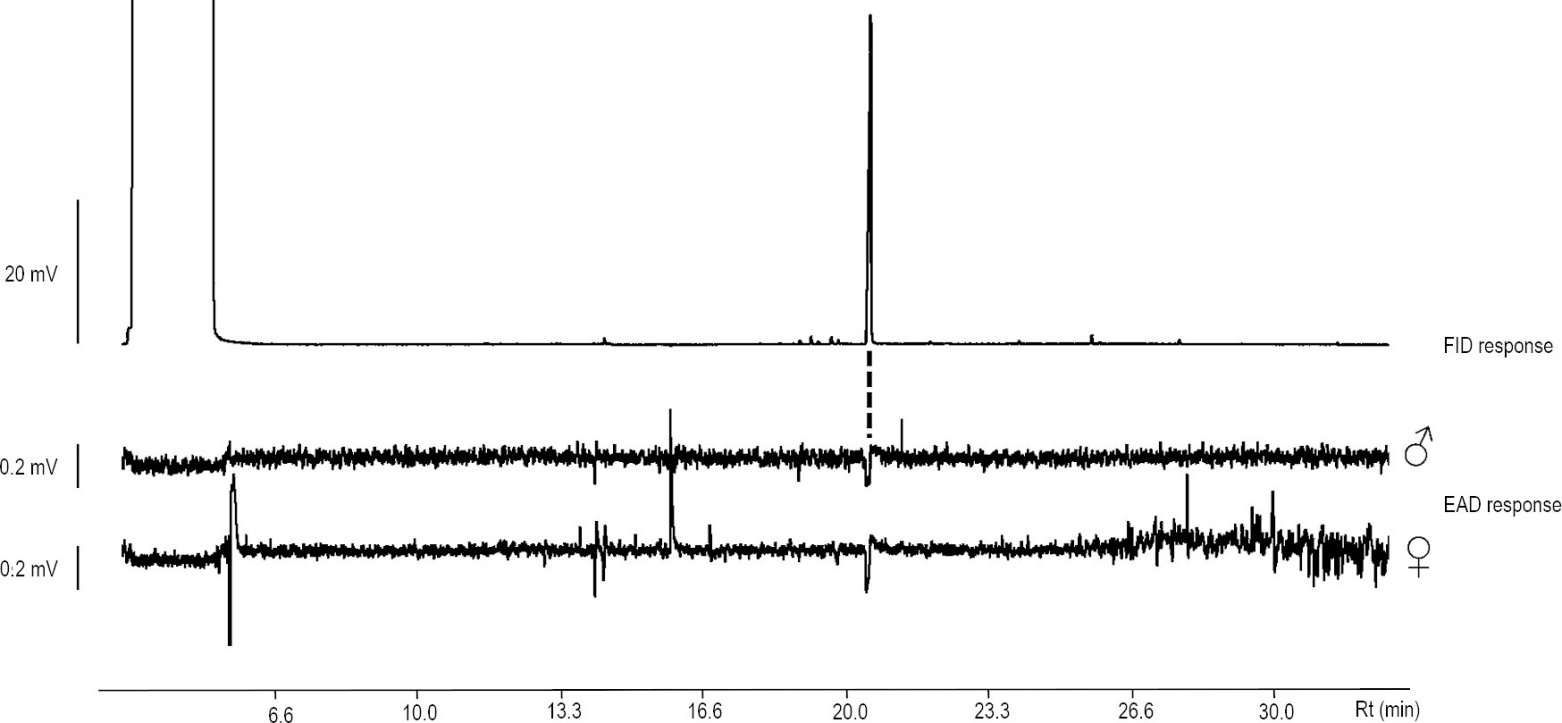
GC-EAD analysis of floral scents extract of *Lagenaria siceraria* by using male and female antenna of Cyclocephalini beetle, *Cyclocephala paraguayensis*, as sensing elements. “FID” flame-ionization detection.

Gas chromatography linked to electroantennographic detection (GC-EAD) analysis of airborne volatiles collected from *L*. *siceraria* showed the presence of a prominent, antennally active peak at 20.5 min (Fig 2).

**Fig 3:**
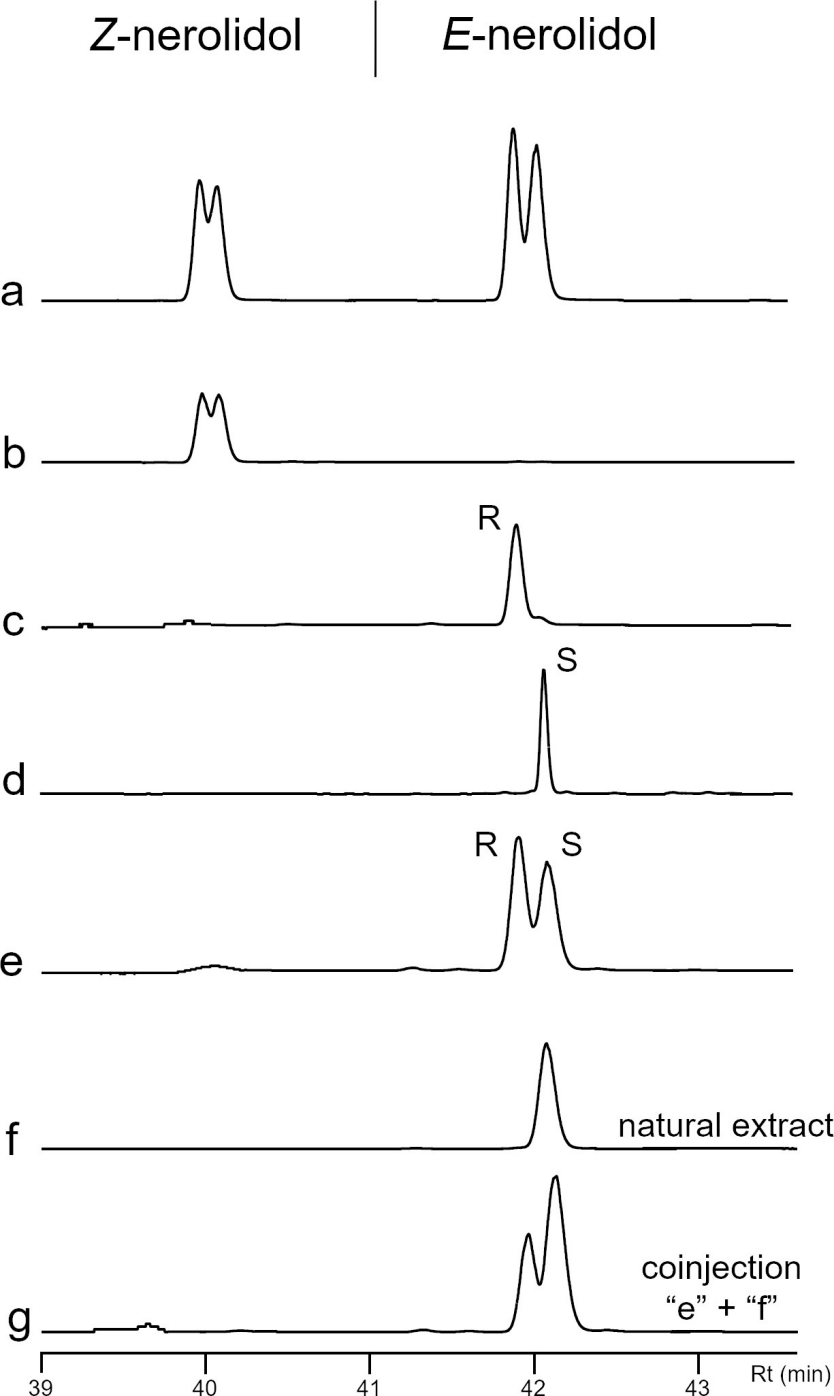
Analysis of bottle gourd flowers, *Lagenaria siceraria*, extract, and synthetic isomers of nerolidol by gas chromatography equipped with a chiral column. a) *Z* and *E*-nerolidol (left to right) isomers of nerolidol (a mix of isomers); b) *Z*-nerolidol isomers; c) Laboratory synthetized of (*3R*,*6E*)-nerolidol (gift from Dr. Robert Hanus, Czech Republic); d) (*E*)-nerolidol from Sigma-Aldrich; e) Mix of 40 ng from each (*3R*,*6E*)-nerolidol and (*3S*,*6E*)-nerolidol; f) Natural extract from VOCs of *L*. *siceraria* flowers; g) Coinjection of “e” and “f” chromatogram extracts.

The correspondent compound was tentatively identified by GC-MS as a nerolidol isomer, which led us to conduct *cis* and *trans* identification followed by chiral analysis. The EAD active compound was then identified as *trans-*nerolidol (i.e., (6*E*)-nerolidol, IR = 1568) by comparing the mass spectra and retention indexes of the natural product and authentic standards (Fig 3A–3F). Lastly, chiral analysis using nerolidol standards led to the full characterization of the natural product as (*3S*,*6E*)-nerolidol (Fig 3G).

Quantification of nerolidol from male and female flowers showed that this VOC comprises 97% of the volatile bouquet and was released at the rate of ~1.3 μg/hour under field conditions.

### Field evaluation of synthetic nerolidol

In our first field trial, a total of six adults of Cyclocephalini beetle, *C*. *paraguayensis*, landed on nerolidol-treated dummies, whereas no beetle was observed on control dummies (two-tailed Exact Binomial test; *P* = 0.031).

**Fig 4:**
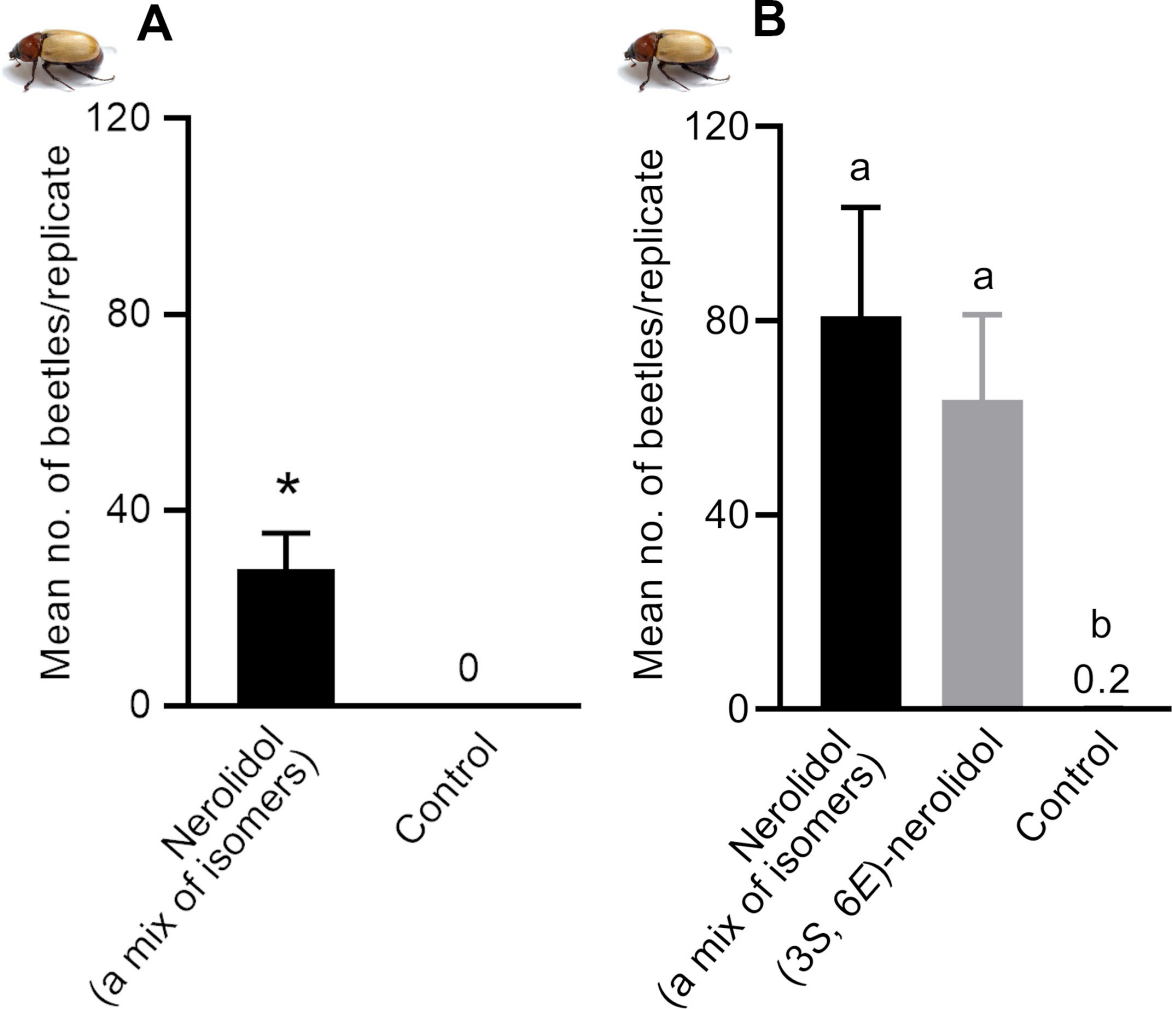
Mean number of Cyclocephalini beetle, *Cyclocephala paraguayensis*, caught per replicate in field experiments conducted in Cassilândia, MS, Brazil. Treatments: Nerolidol (a mix of isomers) = mixture of four isomers of nerolidol; (3*S*, 6*E*)-nerolidol = synthetic nerolidol, which is identical to the stereoisomer of nerolidol produced by *L*. *siceraria* flowers. A) Mean number of adults of *C*. *paraguayensis* caught per replicate of nerolidol-baited traps and controls in the field trial of 2018. *Treatment is significantly different at *P* < 0.05 according to Exact binomial test goodness-of-fit; B) Mean number of adults of *C*. *paraguayensis* caught by traps containing synthetic nerolidol isomers in the field trial of 2019. Treatments had a significant effect on attraction of Cyclocephalini beetles (Friedman’s Q_2,30_ = 20.002, *P* < 0.001; n = 10 replicates). Means followed by same letter are not significantly different (REGWQ test, *P* > 0.05).

In the second field experiment, we evaluated the response of adults of *C*. *paraguayensis* to nerolidol-baited traps in two different locations: Cassilândia, State of Mato Grosso do Sul and Piracicaba, State of São Paulo. In both locations, traps baited with nerolidol (a mix of isomers) captured significantly more beetles than control traps. In Cassilândia, 280 adults of *C*. *paraguayensis* were captured by nerolidol-baited traps during the tests, with no beetles captured in control traps (two-tailed Exact Binomial test; *P* < 0.001; Fig 4A). Although total captures in Piracicaba (n = 32 adults) were lower than in Cassilândia, nerolidol-baited traps captured significantly more beetles than control traps (two-tailed Exact Binomial test*; P* < 0.001). The sex ratio of captured beetles did not differ significantly from the 1:1 ratio in both Cassilândia (51.4% females; 95% Clopper-Pearson exact confidence interval: 0.454–0.574; *P* = 0.632) and Piracicaba (41.4% females; 95% Clopper-Pearson exact confidence interval: 0.235–0.610; *P* = 0.353).

In the third field tests, we compared captures of *C*. *paraguayensis* in traps baited with nerolidol (a mix of isomers), (3*S*,6*E*)-nerolidol, and control traps. Throughout these experiments, a total of 1,146 beetles were captured in nerolidol traps (i.e., traps baited with either enantiopure nerolidol or a mix of isomers). By contrast, only two individuals (males) were caught in control traps. Although treatment and control were significantly different (Q_2,30_ = 20.002; *P* < 0.001; Fig 4B), there was no statistical difference in catches by the mix of isomers or (3*S*,6*E*)-nerolidol (Fig 4B). We, therefore, concluded that the other isomers were neither attractive nor inhibitors. Of note, the sex ratio of the captured beetles in the two nerolidol treatments were somewhat female-biased (mix of isomers = 54.4% female, 95% Clopper-Pearson exact confidence interval: 0.509–0.578; *P* = 0.012; and (3*S*,6*E*)-nerolidol = 58.4% female, 0.544–0.622; *P* < 0.0001). However, as we did not measure the sex ratio of the field populations while conducting bioassays, it is not possible to conclude that traps captured significantly more female than male beetles.

## Discussion

Our results suggest that (3*S*,6*E*)-nerolidol mediates aggregation of the Cyclocephalini beetle, *C*. *paraguayensis*, on bottle gourd flowers, *L*. *siceraria*. We showed that this sesquiterpene alcohol represents the major VOC emitted by the floral scents of *L*. *siceraria*, and field tests demonstrated that nerolidol-baited traps captured significantly more beetles than control traps. Thus, the most prominent compound in bottle gourd floral scent is sufficient for the attraction of *C*. *paraguayensis*, which corroborates similar scenarios found in chemical ecology studies involving other anthophilous Cyclocephalini beetles [8, 10, 12, 13].

Surprisingly, field tests showed captures in traps baited with racemic nerolidol (a mix of four isomers) did not differ significantly from those in traps having the enantiopure nerolidol as a lure. Thus, the three other non-natural isomers of nerolidol are neither attractant nor deterrent.

In the second field experiment, the variation on the beetle catches between Piracicaba and Cassilândia might have been due to the differences in the abundance of *C*. *paraguayensis* in these two locations. Piracicaba comprises the biome Atlantic Rain Forest, which has been observed to harbor small and scattered populations of *C*. *paraguayensis*. Conversely, Cassilândia is located in the biome Cerrado, which appears to sustain a higher population of this species [22].

We, therefore, concluded that C. *paraguayensis* rendezvous in *L*. *siceraria* is mediated by nerolidol, which promotes mating opportunities. There is no evidence in the literature indicating that chemical communication in *C*. *paraguayensis* involves sex pheromones. If it does, a long-range aggregation-sex pheromone (sensu Cardé 2014 [23]) is probably unnecessary for sexual communication, as nerolidol mediates aggregation of both sexes. It is conceivable, however, that once *C*. *paraguayensis* beetles are brought together on flowers, species and/or sex recognition may take place by morphological traits [24], and/or by a hitherto unknown short-range, contact semiochemicals as cuticular hydrocarbons [25].

In this study, we showed that *C*. *paraguayensis* also uses bottle gourd flowers of *L*. *siceraria* for nourishment given that pollen grains were found in the gut epithelium of males and females. The energy-rich pollen grains may increase the fitness of these Cyclocephalini beetles for mating and oviposition [4].

Our findings do not provide evidence for a mutualistic insect-plant relationship, as previously suggested for other anthophilous cyclocephalines [10, 12]. The floral biology traits of cucurbits seem to be more suitable for pollination by bees [26], and flowers are particularly successful pollinated by hawkmoths [21]. This fact corroborates with the presence of nerolidol in the floral scents of bottle gourd, which is a compound commonly related to sphingophilous flowers [27] and thereby accounted to belong to “white-floral image” in moth-pollinated taxa [28]. To date, *C*. *paraguayensis* has been registered only visiting flowers of *Kielmeyera variabilis* Mart. & Zucc.(Calophyllaceae) and *Bauhinia curvula* Benth. (Fabaceae) [15, 18], which are not cantharophilous species. Thus, it is unlikely that the relationship of *C*. *paraguayensis* with these flowers is specific. Besides, floral preferences by *Cyclocephala* species may reflect in gregarious distribution; once *C*. *paraguayensis* beetles are widely distributed in Brazil [22], we surmise they exploit broader odor components than other *Cyclocephala* species with mutualistic floral interaction. Possibly, *C*. *paraguayensis* represents a case of beetle acquiring preference for certain floral odors by its feeding habit during the larval stage; and/or it approaches these flowers when few or no cantharophilous species are in anthesis [15].

Additionally, *C*. *paraguayensis* visits caused damage to *L*. *siceraria* flowers, although the effects of these damages on the development of fruits remain to be investigated. This destructive feeding behavior has been commonly observed in *Cyclocephala* species visiting eudicot flowers [3]. Further research on the chemical ecology of *Cyclocephala* species associated with eudicot flowers is needed to get a better understanding of this interaction. This includes the role of floral volatiles for insect reproduction and/or pollination services, as well as the investigation of *Cyclocephala* beetles volatiles for intraspecific communication.

## Methods

### Sexual and feeding behavior of insects

The behavior of Cyclocephalini beetle, *C*. *paraguayensis*, was observed in a bottle gourd flowers of *L*. *siceraria*, growing area owned by the University of São Paulo, Piracicaba, Brazil (22°42’47.1” S, 47°37’32.7” W). We initially recorded the arrival time of the first beetles on flowers of *L*. *siceraria*, so the observations in the subsequent days started 30 min earlier. Flowers with insects were marked, and the following parameters were evaluated every 30 min from 7:00 p.m. to 11:00 p.m.: sex of visited flowers; the total number of insects landed per flower; and the number of mating couples per flower. We repeated these observations for 4 d in the same *L*. *siceraria* area.

Additionally, a sample of 10 males and females of *C*. *paraguayensis* present within the flowers were taken to verify the presence of pollen in the gut content. Insects were dissected with sharp forceps, and pieces of the midgut and/or the hindgut epithelium were removed and immersed in 0.9% sodium chloride solution. Next, the material was analyzed under a light microscope at 20X magnification. Fresh pollen from *L*. *siceraria* flowers was prepared in glass slides with the saline solution as reference material.

### Sampling floral VOCs

VOCs from *L*. *siceraria* flowers were conducted outdoors in the above-mentioned bottle gourd plantation and the laboratory through dynamic headspace extraction. In the field, VOCs were collected from individual male and female *L*. *siceraria* flowers recognized according to morphological features [20]. These flowers were enclosed within a 17 × 20 cm polyester oven bags (Wyda^®^, Sorocaba, São Paulo, Brazil). Collectors consisted of glass pipets packed with 30 mg of the adsorbent polymer Hayesep^®^ Q (80/100 mesh; Supelco, Bellefonte, PA, USA), which were attached to the bag outlets. Charcoal-filtered air was pushed with a portable pump through the bag and collectors at ~200 mL/min. Bottle gourd flowers were aerated from 7:30 p.m. to 08:30 p.m., which corresponded to the active period of the beetles. Trapped VOCs were eluted from collectors using 0.3 mL of distilled hexane into 2 mL amber vials that were stored at -30°C until analysis. Aerations of empty bags were made in parallel to monitor system contaminants. In the same fashion, VOCs were collected from four detached flowers inside a vertical glass jar (55 cm long × 8 cm id) under laboratory conditions (23 ±2°C; 60 ±10% RH). Collectors attached to the jar outlets were packed with 150 mg of adsorbent polymer. Charcoal-filtered air was pushed through jars and collectors at ~300 mL/min, and collections were made overnight for up 11 h. Trapped VOCs were eluted from collectors using 1.5 mL of distilled hexane into 2 mL amber vial. To use these extracts for GC-EAD analysis, the resulting aliquots were then concentrated to 500 μL under a gentle flow of N_2_.

### GC-EAD recordings

Extracts of VOCs from *L*. *siceraria* flowers were analyzed by GC-EAD, i.e., gas chromatography (Shimadzu GC-2010 gas chromatograph, Shimadzu Corp., Kyoto, Japan) linked to an electroantennographic detector system (EAD, Ockenfels Syntech, Buchenbach, Germany) using *C*. *paraguayensis* antenna as the sensing element. The GC was equipped with a low polarity stationary phase column (30 m × 25 μm × 25 mm; Rtx-5; RESTEK, Bellefonte, PA, USA) connected to a stainless steel crosshead split with two deactivated columns for conducting effluent to FID and EAD detectors at the ratio of 1:3. Helium was used as the carrier gas at a linear velocity of 30.3 cm/s, and 98.3 kPa was set for column head pressure. EAD preparations consisted of male or female excised antennae, and electrodes were made of glass pipette/gold wire filled with a Beadle-Ephrussi Ringer solution (3.75 g NaCl, 0.175 g KCl, 0.14g CaCl_2_.2H_2_O, 500 mL H_2_O). The base of the antenna was inserted into the indifferent electrode, and the tip of antennal lamellae was connected to the recording electrode for signal acquisition (Universal Probe, signal amplification 10x). The antenna preparation was inserted into a glass duct connected to the EAD deactivated column tip, carrying a humidified air stream at 0.3 mL/min. Two microliter aliquots of laboratory-collected volatile extracts were injected in splitless mode at 250°C, and the GC oven was set at 40°C for 5 min and then increased to 250°C at 10°C/min. FID and EAD signals were recorded by Syntech GcEad software (version 4.6).

### Chemical analysis

The EAD active compound in the VOCs was identified by gas chromatography coupled to a mass spectrometer (GC-MS) with a Shimadzu GCQP-2010 Ultra gas chromatograph (Shimadzu Corp., Kyoto, Japan), equipped with a non-polar stationary phase column (30 m × 25μm × 25 mm; Rxi-1MS; RESTEK, Bellefonte, PA, USA). One microliter of the extract was injected at 250°C in split mode (1:3). The GC oven temperature was the same for GC-FID/EAD analysis (above). The helium carrier gas was maintained at a flow of 1.3 mL/min in linear velocity at 41.1 cm/s and 70.7 kPa pressure. Both ion source and quadrupole temperatures were set at 250°C. Mass spectra were recorded in electron impact mode (70 eV) from *m/z* 35–280. Compounds were identified by their retention indexes, mass spectra comparison with Library NIST 11, and injection of authentic standards (Sigma-Aldrich). The target compound was quantified by GC-FID with a Shimadzu GC-2010 gas chromatograph (Shimadzu Corp., Kyoto, Japan) equipped with a non-polar stationary phase column (30 m × 25μm × 25 mm; Rtx-1; RESTEK, Bellefonte, PA, USA) following the same set-up of GC-FID/EAD analysis. We added 3 μL of a 1 mg/mL solution of octadecane as an internal standard in all samples for quantification.

The absolute configuration of flower-produced nerolidol was determined using an Agilent 6890GC with flame ionization detector equipped with a permethylated *β*-cyclodextrin stationary phase chiral column (30 m × 25μm × 25 mm; HP-CHIRAL-20B; J&W Scientific, Folsom, CA, USA). Synthetic nerolidol (a mixture of isomers) and individual geometric isomers were purchased from Sigma-Aldrich to obtain retention time of *cis* and *trans*-nerolidol. A sample of synthetic (*3R*,*6E*)-nerolidol was kindly donated by Dr. Robert Hanus (Institute of Organic Chemistry and Biochemistry. Academy of Sciences of the Czech Republic). All compounds solution in hexane (40 ppm) were injected and detected at 250°C. Initial oven temperature was 85°C for 1 min and then increased to 190°C at 2°C/min. For both chiral identification and field assays, we used the (*E*)-nerolidol from Sigma-Aldrich, which was previously determined to be comprised of almost 100% (*3S*,*6E*)-nerolidol [29], and also confirmed in our GC analysis (Fig 3D).

### Field bioassays of synthetic flower volatiles

We conducted three independent field trials. In the first tests, we mimicked the *L*. *siceraria* flowers by using flower-shaped dummies made from white filter paper, which were impregnated with 100 mg of neat nerolidol (a mixture of isomers, Sigma-Aldrich). Control dummies had no compound. Dummies were attached to wood sticks (25 cm long), which were inserted into the ground, so the dummies were at 23 cm high. Nerolidol-treated and control dummies were randomly distributed in three pairs, with each pair containing one dummy of one treatment. Dummies were spaced 3 m apart, and each pair of dummies was placed ~ 5 m from the other. Bottle gourd flowers dummies were checked for visiting beetles from 06:00 p.m. to 09:00 p.m. Soon after landing, Cyclocephalini beetles were removed from dummies to avoid potential intraspecific attraction. This bioassay was carried out in the same *L*. *siceraria* groove in Piracicaba, SP, Brazil (see above) for 2 d (March 12–13, 2018), with dummies replacement made for each replicate.

The second field tests were conducted in the same area in Piracicaba and a pasture area owned by the Mato Grosso do Sul State University, Cassilândia, MS, Brazil (~ 800 km from Piracicaba; 19°05’35.8” S, 51°48’52.5” W). We used custom-made, cross-vane intercept traps (black polystyrene panels: 18 × 24 cm), with 0.5-L plastic jars attached to the bottom of the trap. Both control and treatment traps were loaded with 200 ml of a solution of water and dish detergent to kill the captured Cyclocephalini beetles. Traps were hung from L-shaped PVC pipe frames (1.5 m long) that were supported by 1-m reinforcing steel bars hammed into the ground. The lures were pieces of filter paper (2 × 6 cm) loaded with 50 mg of neat nerolidol (a mixture of isomers). Control lures were filter papers with no compound. Lures were hung in the central opening slot of traps with a diaper pin 1-h prior to the sunset. Treated and control traps were randomly distributed. Seven even pairs of traps were deployed in Piracicaba and 10 pairs in Cassilândia. Traps were spaced 20 m apart, and pairs of traps were set ~30 m from each other. Traps were serviced for captured beetles in the morning. For every replicate date, the lures were replaced, and the treatment traps were switched in position to avoid positional effects. Overall, the bioassay was repeated for 5 d in Piracicaba (October 17–22, 2018) and for 1 d in Cassilândia (October 1^st^, 2018).

The third bioassay was conducted in the same area in Cassilândia (October 1–2, 2019), which tested the following treatments: (i) 5 mg of neat (3*S*,6*E*)-nerolidol (*trans*-nerolidol, Sigma-Aldrich); (ii) 20 mg of neat nerolidol (a mixture of isomers), considering 5 mg per isomer; and (iii) control (filter paper with no compound). Treatments were assigned randomly to traps within five blocks. Evaluations were carried out every morning for two days. Lures were replaced 1-h prior to the sunset, and treatment traps were changed one position within blocks to control for positional effects.

Captured cyclocephalini specimens were sent to Dr. Paschoal Coelho Grossi (Federal Rural University of Pernambuco—UFRPE) for taxonomy. A voucher specimen of Cyclocephalini beetle, *C*. *paraguayensis*, was deposited in the collection of the Museum of Entomology (#6945), Department of Entomology and Acarology (University of São Paulo, Piracicaba, Brazil). Collection of beetles in the field were conducted under SISBIO permit #60104 and #67772 from the Brazilian Ministry of the Environment. This work was submitted to Genetic Heritage Management Council by SisGen within project registration #AFC4FA2.

### Statistical analysis

Data from experiments 1 and 2 were analyzed with the two-tailed Exact Binomial test to verify whether the ratio of beetles attracted to nerolidol and control differed significantly from the expected 1:1 ratio at 5% probability [30]. The test was performed from a spreadsheet provided by http://www.biostathandbook.com/exactgof.html (accessed January 17, 2019).

Differences between treatment means from experiment 3 were analyzed using the nonparametric Friedman’s Test (PROC FREQ, option CMH; SAS Institute, 2011), because data did not pass the assumptions of ANOVA [31]. Replicates were defined by block and collection dates. Replicates with no capture of *C*. *paraguayensis* in any trap were excluded from analyses. Once an overall significance was detected by the Friedman's test, pairs of means were compared using the REGWQ multiple range test, which controls for type I experiment-wise error rate [32]. For all bioassays, the sex ratio of attracted adults of *C*. *paraguayensis* was compared to a nominal proportion of 0.5 with 95% Clopper-Pearson Exact Confidence Intervals [33].
